# Exploring Human Epidermal Growth Factor Receptor 2 (HER2)-Low Early Breast Cancer in a Moroccan Population: Clinical Characteristics and Survival Outcomes

**DOI:** 10.7759/cureus.90253

**Published:** 2025-08-16

**Authors:** Meryem Maskrout, Farah Boutaagount, Rania Mokfi, Soundous Bennour, Chaymae Senoussi, Safiya Mahlaq, Fadoua Rais, Ghizlane Rais

**Affiliations:** 1 Medical Oncology Department, Faculty of Medicine and Pharmacy of Agadir, Ibn Zohr University, Agadir, MAR; 2 Clinical Research and Epidemiology Department, Faculty of Medicine and Pharmacy of Rabat, Mohammed V University, Rabat, MAR; 3 Radiotherapy Department, University of Montreal Health Centre, Montreal, CAN

**Keywords:** early-stage breast cancer, her2-low breast cancer, prognostic risk factors, real world data, survival outcomes

## Abstract

Breast cancers with low human epidermal growth factor receptor 2 (HER2) expression, classified as an immunohistochemistry (IHC) score of 1+ or 2+ without gene amplification, have recently drawn increased interest due to the emergence of novel anti-HER2 therapies. However, there is still debate over whether HER2-low tumors form a distinct category. This study set out to examine and compare the clinical characteristics and outcomes of early-stage breast cancer patients with HER2-zero and HER2-low expression. We reviewed medical records of stage I-III patients treated at Souss-Massa University Hospital Center in Morocco between 2016 and 2018. HER2-positive cases were excluded, leaving two comparison groups: HER2-zero and HER2-low. Using statistical methods, we assessed baseline characteristics and survival outcomes - disease-free survival (DFS) and overall survival (OS) - through Kaplan-Meier analysis, log-rank testing, and Cox regression models based on key prognostic factors like age, hormone receptor (HR) status, Ki-67 index, and lymph node involvement. Among 322 patients studied, we found 114 were HR-positive/HER2-zero, 95 HR-positive/HER2-low, 85 HR-negative/HER2-zero, and 28 HR-negative/HER2-low. Tumor size, nodal status, and histologic grade were similar across both groups. Notably, HER2-low tumors were more often HR-positive (95 (77.2) vs. 114 (57.3), p < 0.001), and HER2-zero tumors were more likely to have high Ki-67 levels (>20%) (95 (47.73) vs. 41 (33.33), p = 0.009). While certain subgroups, like HR-positive or high Ki-67 cases, showed better DFS in the HER2-low group, overall, there were no significant differences in DFS or OS. These results suggest HER2-low and HER2-zero breast cancers share largely similar features and outcomes; however, in certain subgroups, HER2-low tumors were associated with significantly better DFS, possibly reflecting differences in tumor biology. Further research is essential to clarify the biological role of HER2-low expression.

## Introduction

Breast cancer has become the most commonly diagnosed cancer worldwide, with approximately 2.3 million new cases and 685,000 deaths reported in 2020 [1]. It remains the leading cause of cancer-related illness and death among women globally [2,3]. This disease is marked by significant clinical and molecular diversity, encompassing a wide array of subtypes that vary in their biological behavior, prognosis, and treatment response. This heterogeneity has major implications for clinical care, underscoring the need for personalized treatment strategies tailored to the molecular profile of each tumor [4]. Traditionally, therapeutic decisions in breast cancer have been guided by hormone receptor (HR) and human epidermal growth factor receptor 2 (HER2) status. HER2-positive tumors respond well to targeted anti-HER2 therapies, while HER2-negative cancers have historically been considered unsuitable for these interventions. However, recent developments have challenged this binary framework with the emergence of a new subgroup known as HER2-low, defined by an immunohistochemistry (IHC) score of 1+ or 2+ and a negative in situ hybridization (ISH) test [5,6]. Interest in the HER2-low category has grown following promising results from new antibody-drug conjugates (ADCs), particularly trastuzumab deruxtecan (T-DXd), which demonstrated notable clinical benefits in patients with HER2-low metastatic breast cancer, as shown in the DESTINY-Breast04 trial [7]. These findings have prompted a reassessment of how HER2 status is classified and sparked a growing interest in the biological and clinical features of this emerging subgroup. Although HER2-low tumors exhibit some unique biological features, it remains uncertain whether they represent a distinct clinical entity [4,8]. The discussion so far has focused more on the treatment implications than on the intrinsic biology of HER2-low tumors [5]. As a result, there is a growing need to better define the clinicopathological characteristics of HER2-low cancers, especially in diverse populations and early-stage disease settings, in order to improve prognostic assessments and guide treatment decisions. In this context, studying a Moroccan population is particularly relevant, as potential regional variations in tumor biology, genetic background, and differences in access to diagnostic and therapeutic resources may influence the prevalence, biological behavior, and prognostic impact of HER2-low breast cancer. Against this backdrop, our retrospective study aims to investigate and compare the clinicopathological features and prognostic outcomes of HER2-low and HER2-zero tumors in early-stage breast cancer.

## Materials and methods

Patient data

This retrospective study was conducted at the Department of Medical Oncology, Souss-Massa University Hospital Center in Agadir, Morocco. It included women diagnosed with early-stage invasive breast cancer (stages I to III) between January 2016 and December 2018. Patients were identified through the hospital’s electronic database. To be included, patients needed to have a confirmed histological diagnosis of invasive breast carcinoma, available HER2 IHC results, and complete clinical, pathological, and follow-up information. Exclusion criteria were HER2-positive disease (defined as IHC 3+ or IHC 2+ with a positive ISH result), metastatic cancer at diagnosis, or ductal carcinoma in situ (DCIS) without invasion. The study complied with the ethical standards outlined in the Declaration of Helsinki (1964) and was approved by the local ethics committee.

Clinical data

Clinical information was gathered from archived medical records. Variables collected included age, menopausal status, body mass index (BMI), TNM staging, histological type (ductal or lobular), tumor grade (based on the Scarff-Bloom-Richardson (SBR) classification), HR status, and the Ki-67 proliferation index. Treatment-related data were also documented, including the type of surgery (breast-conserving vs. mastectomy), use of chemotherapy (adjuvant, neoadjuvant, or none), radiotherapy, and hormone therapy.

Histopathological data

Pathology reports provided detailed histopathological features, including estrogen receptor (ER) and progesterone receptor (PR) status, as well as HER2 status. HR positivity was defined as nuclear staining in at least 1% of tumor cells for ER and/or PR. Tumors lacking both were classified as HR-negative [9]. HER2 status was assessed via IHC by multiple experienced pathologists within our region and interpreted in line with the 2023 American Society of Clinical Oncology/College of American Pathologists (ASCO/CAP) guidelines, without central pathology review. Tumors that were IHC 3+ or IHC 2+ with ISH amplification were deemed HER2-positive and excluded. HER2-low was defined as IHC 1+ or 2+ with negative ISH, while HER2-zero corresponded to IHC 0 [10].

Treatment modalities

Treatment information was obtained from medical records and included surgical approach, chemotherapy, radiotherapy, and endocrine therapy. Surgery was either breast-conserving or mastectomy with axillary node dissection. Chemotherapy could be given before or after surgery, or not at all, based on tumor features and decisions from multidisciplinary meetings. Radiotherapy followed institutional protocols and was applied post-surgery when indicated. Hormone therapy, such as tamoxifen or aromatase inhibitors, was prescribed according to HR expression and menopausal status.

Statistical analysis

Data were analyzed using IBM SPSS Statistics for Windows, Version 26 (Released 2020; IBM Corp., Armonk, New York, United States). Descriptive statistics summarized the clinical and pathological features. Comparisons between the HER2-low and HER2-zero groups were done using Chi-square or Fisher’s exact test for categorical data and Student’s t-test or Mann-Whitney U test for continuous data. Disease-free survival (DFS) and overall survival (OS) were estimated using Kaplan-Meier curves, with group differences assessed by log-rank tests. Univariate Cox regression models analyzed the effect of HER2 status on DFS and OS. These analyses were stratified by key prognostic factors: age, HR status, Ki-67 index, and nodal involvement. HER2-zero served as the reference group. Hazard ratios (HRs) and 95% confidence intervals (CIs) were reported, and p-values under 0.05 were considered statistically significant.

## Results

Demographic and clinical characteristics

The study included a total of 322 women diagnosed with localized breast cancer. These patients were categorized into two groups based on HER2 expression: HER2-zero: 199 (61.8) and HER2-low: 123 (38.2). The average age across the entire cohort was 52.1 ± 12.4 years, with no significant age difference between the groups (p = 0.173). While not statistically significant, there was a trend toward a higher proportion of postmenopausal women in the HER2-low group (p = 0.069). BMI was similarly distributed in both groups, with no meaningful differences observed (p = 0.851) (Table 1).

**Table 1:** Clinicopathological characteristics of patients with early-stage HER2-low and HER2-zero breast cancer The data are expressed using N values (%) and mean ± SD. ^1^ Student's t-test was used for continuous variables. ^2^ Categorical variables were compared using the chi-square test. HER2: human epidermal growth factor receptor 2; HR: hormone receptor; BMI: body mass index; IDC: invasive ductal carcinoma; ILC: invasive lobular carcinoma; SBR: Scarff-Bloom-Richardson grade; Ki-67: proliferation index; BCS: breast-conserving surgery; BRS: mastectomy (breast radical surgery); RT: radiotherapy; T: tumor size (TNM classification); N: nodal status (TNM classification)

Variables	All patients (n=322)	HER2-0 (n=199)	HER2-low (n=123)	Test	P-value
Age	52.1 ± 12.4	50.9 ± 11.7	52.8 ± 12.7	1.37¹	0.173
Menopausal status				3.30²	0.069
Premenopause	182 (56.7)	105 (32.7)	77 (24)		
Postmenopause	139 (43.3)	94 (29.3)	45 (14)		
BMI				0.792²	0.851
<18	17 (6.3)	9 (3.3)	8 (3)		
18-25	99 (36.5)	55 (20.3)	44 (16.2)		
25-30	105 (38.7)	62 (22.8)	43 (15.9)		
>30	50 (18.5)	26 (9.6)	24 (8.9)		
HR				13.3²	<0.001
HR-positive	209 (64.9)	114 (35.4)	95 (29.5)		
HR-negative	113 (35.1)	85 (26.4)	28 (8.7)		
T				1.62²	0.654
T1	67 (20.8)	37 (11.5)	30 (9.3)		
T2	148 (46)	93 (28.9)	55 (17.1)		
T3	42 (13.1)	27 (8.4)	15 (4.7)		
T4	65 (20.1)	42 (13)	23 (7.1)		
N				0.477²	0.490
N0	144 (44.7)	86 (26.7)	58 (18)		
N+	178 (55.3)	113 (35.1)	65 (20.2)		
SBR grade				1.73²	0.422
1	7 (2.2)	4 (1.3)	3 (0.9)		
2	185 (58)	109 (34.2)	76 (23.8)		
3	127 (39.8)	84 (26.3)	43 (13.5)		
Histological type				0.004²	0.998
IDC	291 (90.9)	179 (55.9)	112 (35)		
ILC	21 (6.6)	13 (4.1)	8 (2.5)		
Other	8 (2.5)	5 (1.6)	3 (0.9)		
Vascular emboli				0.006²	0.938
Yes	134 (42)	82 (25.7)	52 (16.3)		
No	185 (58)	114 (35.7)	71 (22.3)		
Ki-67				6.92²	0.009
≤20%	72 (34.6)	37 (17.8)	35 (16.8)		
>20%	136 (65.4)	95 (45.7)	41 (19.7)		
Breast surgery				1.50²	0.221
BCS	104 (32.8)	59 (18.6)	45 (14.2)		
BRS	213 (67.2)	136 (42.9)	77 (24.3)		
Chemotherapy				0.571²	0.450
Neoadjuvant	136 (42.2)	85 (26.2)	51 (16)		
Adjuvant	155 (48.6)	99 (31)	56 (17.6)		
No	31 (9.6)	16 (5)	15 (4.6)		
Endocrine therapy				11.6²	<0.001
Yes	209 (64.9)	114 (35.4)	95 (29.5)		
No	113 (35.1)	85 (26.4)	28 (8.7)		
Adjuvant RT				0.336²	0.562
Yes	267 (85.8)	164 (52.7)	103 (33.1)		
No	44 (14.2)	25 (8)	19 (6.2)		

Tumor characteristics

HR status differed significantly between the groups. HR-positive tumors were notably more common in the HER2-low group compared to HER2-zero (95 (77.2) vs. 114 (57.3); p < 0.001). However, tumor size (T stage) and lymph node involvement (N stage) were similar in both groups (p = 0.654 and p = 0.923, respectively). Histologic grade, as assessed by the SBR system, also showed no significant variation (p = 0.422). Most patients in both groups had invasive ductal carcinoma (IDC), and there was no difference in the distribution of tumor subtypes (p = 0.998). Likewise, the presence of vascular emboli did not differ significantly between the groups (p = 0.938) (Table 1).

Tumor proliferation

Analysis of Ki-67 expression, a marker of tumor cell proliferation, revealed a significant difference. Fewer tumors in the HER2-low group had high Ki-67 levels (>20%) compared to the HER2-zero group (p = 0.009), indicating that HER2-low tumors may exhibit less aggressive biological behavior (Table 1).

Treatment modalities

When comparing treatment approaches, no significant differences were found between the groups regarding the type of breast surgery performed (breast-conserving vs. mastectomy), the use of radiotherapy, or the type of chemotherapy administered (neoadjuvant, adjuvant, or none), with all p-values exceeding 0.05 (Table 1).

Survival outcomes

**Figure 1:**
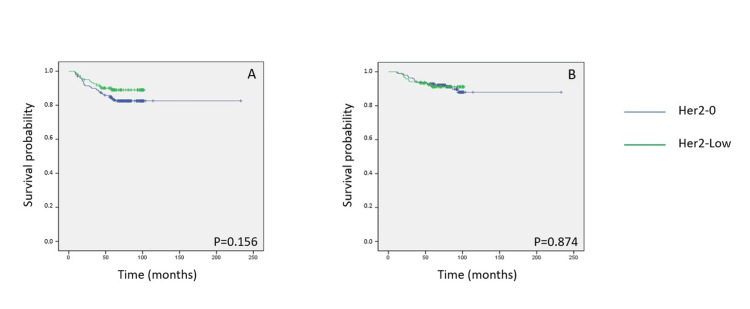
Kaplan-Meier survival curves illustrating five-year disease-free survival (DFS) and overall survival (OS) according to HER2 expression status in patients with early-stage breast cancer. (A, B) DFS and OS for the entire study population comparing HER2-low and HER2-0 groups. HER2: human epidermal growth factor receptor 2

The Kaplan-Meier method was used to evaluate and compare DFS and OS between patients with HER2-low and HER2-zero breast cancer. Over a median follow-up period of 77 months, there was no statistically significant difference in five-year DFS between the two groups (log-rank p = 0.156). Specifically, 34 cases of cancer recurrence were noted in the HER2-zero group, compared to 13 in the HER2-low group. Similarly, OS at five years did not differ significantly between the groups (log-rank p = 0.874), with 18 deaths recorded in the HER2-zero group and 10 in the HER2-low group (Figures 1A-1B).

To better understand how HER2 status might relate to survival outcomes, we carried out further analyses by dividing patients into subgroups based on HR status and tumor cell proliferation (Ki-67 index).

HR-positive patients

**Figure 2:**
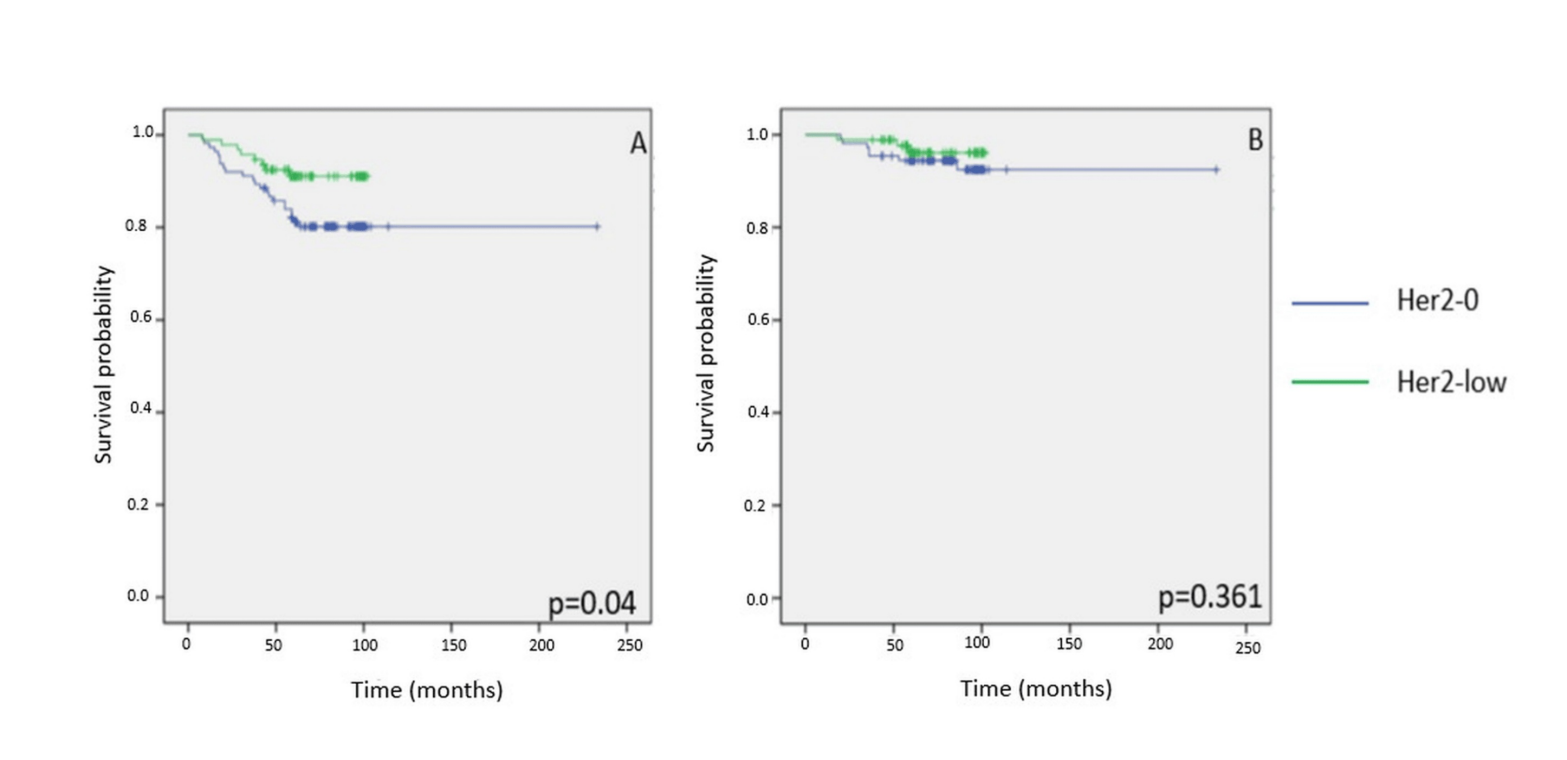
Kaplan-Meier survival curves illustrating five-year disease-free survival (DFS) and overall survival (OS) according to HER2 expression status in patients with early-stage breast cancer. (A, B) DFS and OS among hormone receptor-positive (HR+) patients according to HER2 status. HER2: human epidermal growth factor receptor 2

In patients whose tumors expressed HRs, DFS at five years was significantly better in the HER2-low group compared to the HER2-zero group (log-rank p = 0.04). However, OS did not differ meaningfully between these groups (log-rank p = 0.361). The number of recurrences and follow-up times were comparable in both cases (Figures 2A-2B).

HR-negative patients

**Figure 3:**
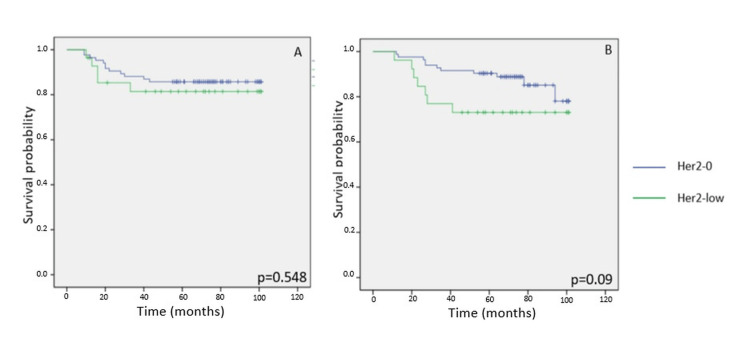
Kaplan-Meier survival curves illustrating five-year disease-free survival (DFS) and overall survival (OS) according to HER2 expression status in patients with early-stage breast cancer. (A, B) DFS and OS among hormone receptor-negative (HR-) patients according to HER2 status. HER2: human epidermal growth factor receptor 2

For those with HR-negative tumors, no significant difference in five-year DFS was found between HER2-low and HER2-zero groups (log-rank p = 0.548). Similarly, OS outcomes were statistically similar (log-rank p = 0.09), and event rates remained low in this subgroup (Figures 3A-3B).

Patients with high proliferation (Ki-67 >20%)

**Figure 4:**
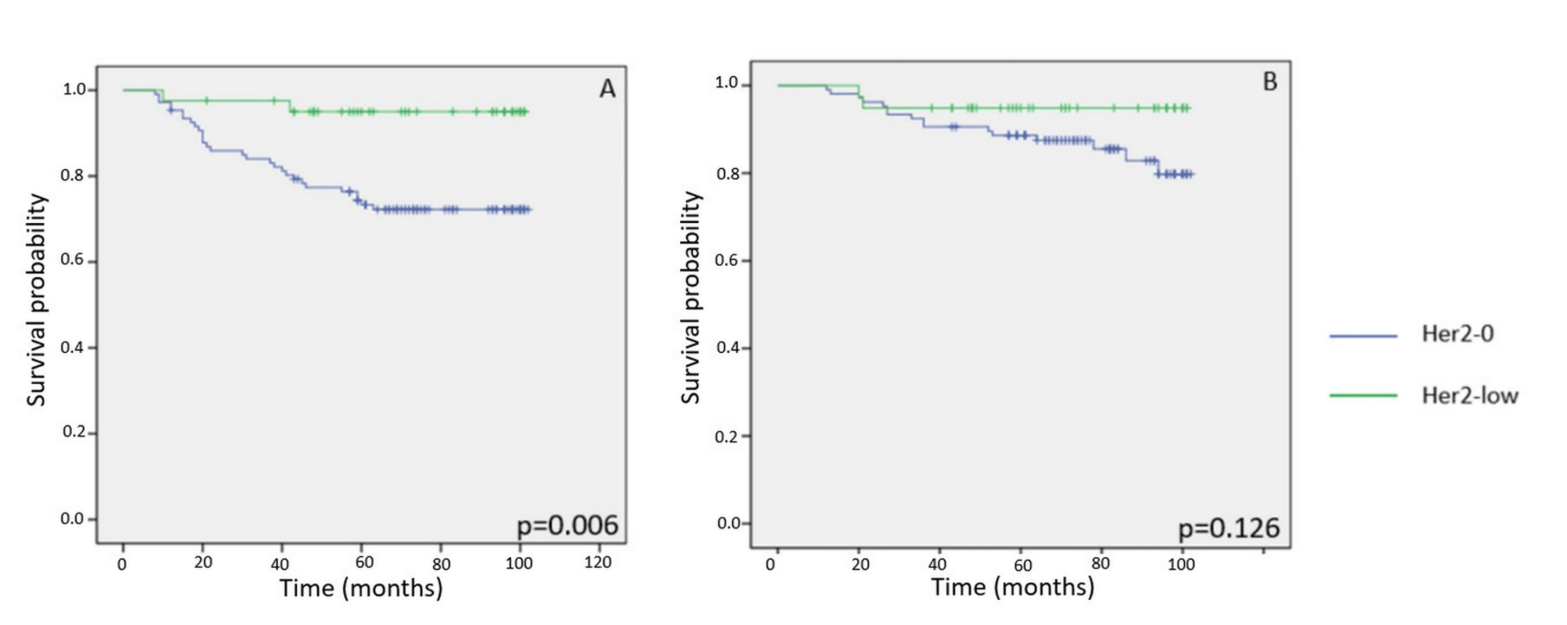
Kaplan-Meier survival curves illustrating five-year disease-free survival (DFS) and overall survival (OS) according to HER2 expression status in patients with early-stage breast cancer. (A-B) DFS and OS in patients with high proliferative tumors (Ki-67 >20%) according to HER2 status. HER2: human epidermal growth factor receptor 2

Among patients whose tumors had a Ki-67 index above 20%, the HER2-low group had significantly better DFS at five years than the HER2-zero group (log-rank p = 0.006). Nonetheless, five-year OS remained statistically similar between the groups (log-rank p = 0.126), and both had similar numbers of censored data points (Figures 4A-4B).

Cox regression subgroup analyses

Further insights came from Cox regression models, which explored the prognostic effect of HER2 status in various subgroups (Table 2).

**Table 2:** Univariate analysis of prognostic factors associated with disease-free survival and overall survival in patients with HER2-low and HER2-zero breast cancer. HER2: human epidermal growth factor receptor 2; HR^1^: hormone receptor; DFS: disease-free survival; OS: overall survival; HR^2^ (Cox): hazard ratio; CI: confidence interval

Variable	DFS			OS		
	HR^2^ (HER2-low vs. HER2-zero)	95% CI	p-value	HR^2^ (HER2-low vs. HER2-zero)	95% CI	p-value
Age ≤50	1.042	0.47-2.29	0.919	0.566	0.21-1.46	0.242
Age >50	3.370	0.99-11.44	0.051	5.526	0.70-43.19	0.103
HR^1^- negative	0.727	0.25-2.06	0.550	0.485	0.18-1.25	0.136
HR^1^- positive	2.250	1.00-5.05	0.049	1.837	0.47-7.11	0.379
Ki-67 ≤20%	1.814	0.16-20.01	0.627	0.932	0.05-14.90	0.960
Ki-67 >20%	5.901	1.40-24.73	0.015	3.150	0.72-13.72	0.126
N0	45.25	0.23-8773.8	0.156	1.965	0.396-9.75	0.409
N+	1.034	0.52-2.03	0.923	0.819	0.33-2.00	0.662

Disease-free survival (DFS)

In women over 50 years old, HER2-low status was linked to a higher risk of recurrence compared to HER2-zero (HR = 3.370; 95% CI: 0.99-11.44; p = 0.051), a result that approached statistical significance. For HR-positive patients, those with HER2-zero tumors had significantly poorer DFS than those with HER2-low tumors (HR = 2.250; 95% CI: 1.00-5.05; p = 0.049). Interestingly, in patients with high Ki-67 expression (>20%), HER2-low tumors were also associated with a significantly higher recurrence risk (HR = 5.901; 95% CI: 1.40-24.73; p = 0.015).

No significant DFS differences were seen in women aged 50 or younger (p = 0.919), in HR-negative patients (p = 0.550), in those with low Ki-67 expression (≤20%) (p = 0.627), or based on lymph node status (N0: p = 0.156; N+: p = 0.923).

Overall survival (OS)

No subgroup showed a statistically significant difference in OS between HER2-low and HER2-zero. The highest HR was in women over 50 (HR = 5.526; 95% CI: 0.70-43.19; p = 0.103), but this did not reach significance. Higher, though non-significant, HRs were also seen in HR-positive patients (HR = 1.837), in those with high Ki-67 (HR = 3.150), and in node-negative patients (HR = 1.965), with all p-values above 0.05.

## Discussion

In recent years, the traditional binary classification of breast cancer as either HER2-positive or HER2-negative has been increasingly questioned, especially in light of new evidence supporting the effectiveness of HER2-targeting ADCs in patients with metastatic HER2-low breast cancer [7,11]. Genomic studies, including PAM50-based analyses, have highlighted molecular distinctions between HER2-low and HER2-zero tumors, raising the possibility that HER2-low breast cancer might represent a biologically unique subgroup within HER2-negative disease [11].

This study adds to ongoing efforts to understand the clinical and biological features of HER2-low breast cancer, aiming to optimize treatment strategies. We examined the clinical and pathological profiles, as well as survival outcomes, of early-stage HER2-low breast cancer in a Moroccan patient population, to better characterize this group and compare it to HER2-zero tumors in our local setting.

Our analysis first focused on the differences in clinicopathological characteristics between HER2-low and HER2-zero tumors, followed by survival analyses stratified by major clinical and biological factors.

Age appears to influence the behavior of HER2-low tumors. Although the average age at diagnosis did not differ significantly between groups (52.8 vs. 50.9 years; p = 0.173), subgroup analysis revealed that patients over 50 with HER2-low tumors had a higher recurrence risk (HR = 3.370; 95% CI: 0.99-11.44; p = 0.051), a result that nearly reached statistical significance. This may suggest a role of age-related hormonal factors. Other studies have also noted that HER2-low tumors in HR-positive cases are more often diagnosed in younger women, while in HR-negative subtypes, especially triple-negative breast cancer, they are more frequently seen in older patients [12-15].

In our cohort, 77.23% of HER2-low tumors were HR-positive, consistent with previous findings reporting HR positivity rates between 64% and 88% [11,16]. This supports the view that HER2-low breast cancer is not a uniform entity but spans a biological spectrum, often overlapping with luminal-like subtypes. These luminal tumors, driven by estrogen and progesterone signaling, generally grow more slowly and respond well to endocrine therapy. The similarity between HR+/HER2-low and HR+/HER2-zero tumors suggests that HER2-low status in HR-positive settings may not reflect a biologically separate entity, but rather a variant in HER2 protein expression that doesn’t significantly alter oncogenic signaling [17]. This view is backed by transcriptomic analyses showing that most HR+/HER2-low tumors fall into luminal A or B subtypes on the PAM50 scale [9]. From a prognostic standpoint, our results showed that HER2-zero tumors had significantly worse DFS compared to HER2-low tumors in the HR-positive group (HR = 2.250; 95% CI: 1.00-5.05; p = 0.049). This suggests that HER2-low status may be a potentially favorable prognostic marker in this subgroup. These findings are in line with a recent meta-analysis of over 50,000 patients, which reported improved DFS in HR-positive HER2-low tumors (HR = 0.85; 95% CI: 0.76-0.96; p = 0.007) [18]. Similarly, a significantly better five-year DFS has been reported in HR-positive HER2-low early breast cancer compared to HER2-zero (HR = 0.31; 95% CI: 0.13-0.75; p = 0.01), reinforcing the hypothesis that HER2-low tumors may exhibit less aggressive behavior in this subgroup [19]. In contrast, our data did not show significant differences in OS between HR+/HER2-low and HER2-zero tumors (HR = 1.837; p > 0.05), in line with previous meta-analytic findings reporting no clear OS advantage in this subgroup (HR 0.81; 95% CI 0.66-0.98; p = 0.03) [20]. These trends suggest potential benefit, but confirm the need for future prospective studies.

When examining tumor proliferation, our study found that high Ki-67 expression (>20%) occurred significantly less often in HER2-low tumors compared to HER2-zero tumors (53.95% vs. 71.97%, p = 0.009). This lower frequency points to reduced cellular proliferation in the HER2-low group, indicating a generally less aggressive tumor biology. These results align with recent research that links HER2-low breast cancers with more favorable histological features, such as lower tumor grade and reduced proliferation [19,21]. Ki-67 is a well-established marker of tumor cell proliferation and is considered a key prognostic indicator in breast cancer, particularly within HR-positive subtypes. In these cases, high Ki-67 levels are associated with increased relapse risk and varying responses to systemic treatment [22]. The lower Ki-67 levels seen in our HER2-low group likely reflect a biologically less aggressive tumor type, supporting the theory that HER2-low cancers, especially those driven by hormone signaling, tend to fall within luminal A or B subtypes, which are known for slower growth rates [17,19]. However, while reduced proliferation could suggest a more favorable prognosis, several studies have noted that this does not always lead to better survival outcomes, especially in early-stage disease [21]. This is consistent with our findings, as we found no significant differences in OS between HER2-low and HER2-zero tumors in any of the subgroups studied. Interestingly, when we looked specifically at patients with high Ki-67 expression (>20%), HER2-low tumors were paradoxically linked to a significantly higher risk of recurrence (HR = 5.901; 95% CI: 1.40-24.73; p = 0.015). However, this result should be interpreted with caution, as the small number of events and missing Ki-67 data may have contributed to wide CIs and unstable estimates. Nevertheless, this observation underscores the potential heterogeneity within the HER2-low group and suggests that high Ki-67 HER2-low tumors may represent a distinct and potentially more aggressive biological phenotype, warranting further investigation.

Regarding lymph node involvement, we found no significant differences between HER2-low and HER2-zero groups (N0/N+: p = 0.923). This indicates that low HER2 expression is not necessarily linked to increased nodal spread, contradicting assumptions about more aggressive behavior. These results align with other studies that found no relationship between HER2-low status and regional disease extent. For example, a recent study involving 1,627 patients found no difference in nodal status after matching, but reported better recurrence-free survival in node-positive HER2-low cases (p = 0.033) [21]. Similarly, in our dataset, nodal status was not a significant factor in DFS or OS between groups (DFS: N0: p = 0.156; N+: p = 0.923; OS: N0: HR = 1.965). A large 2024 meta-analysis of 49,763 patients also found improved DFS (HR = 0.83; 95% CI: 0.75-0.92; p = 0.0002) and OS (HR = 0.72; 95% CI: 0.61-0.85; p < 0.0001) in HER2-low tumors compared to HER2-zero, but mainly within HR-positive subgroups [18]. Together, these findings suggest that while lymph node status remains an important prognostic factor, HER2-low expression does not independently predict nodal spread or survival. Current literature further supports that HER2-low tumors are often HR-positive and tend to display milder clinical behavior, emphasizing the need to interpret HER2 status within the broader clinical context [23].

This study has several limitations. Its retrospective, single-center design may introduce selection bias and limit how broadly the results can be applied. Although the overall sample size was adequate, certain subgroups (like HR-negative or node-negative patients) were relatively small, weakening statistical power. Additionally, HER2 status was assessed via local IHC without centralized review, potentially leading to interpretation variability, especially in borderline HER2-low cases. Furthermore, molecular profiling tools such as PAM50 or RNA sequencing were not used, preventing deeper insight into intrinsic tumor biology. Lastly, while the follow-up period was sufficient for DFS evaluation, it may not have been long enough to detect significant differences in long-term OS.

## Conclusions

In this retrospective cohort study focusing on Moroccan patients with early-stage breast cancer, we did not observe any significant differences in tumor size, histologic grade, lymph node involvement, or OS between HER2-low and HER2-zero tumors in the general population. However, HER2-low status was notably linked to HR positivity and lower proliferative activity, both characteristics commonly associated with a less aggressive disease profile. Within the HR-positive subgroup, HER2-low tumors demonstrated significantly better DFS compared to HER2-zero tumors, suggesting that HER2-low expression may be associated with favorable DFS in this context; however, these findings are preliminary and require confirmation in prospective studies.

Despite this, no significant differences in OS were found across any subgroup comparisons. These results underscore the biological heterogeneity of HER2-low breast cancer and emphasize the importance of interpreting HER2 expression in conjunction with broader clinicopathological factors. To better define the prognostic and biological role of HER2-low status, particularly in HR-positive disease, future research should prioritize prospective, multicenter studies incorporating molecular profiling.
